# Development and evaluation of the Measure of the International Learning Environment Status (MILES) in international higher education

**DOI:** 10.1371/journal.pone.0288373

**Published:** 2023-08-17

**Authors:** Xiaoming Xu, Johanna Schönrock-Adema, Nicolaas A. Bos

**Affiliations:** 1 Wenckebach Institute for Education and Training, University of Groningen, University Medical Center Groningen, Groningen, the Netherlands; 2 International Institute of Medicine, Zhejiang University, Yiwu, Zhejiang, China; 3 The Fourth Affiliated Hospital, Zhejiang University School of Medicine, Yiwu, Zhejiang, China; Tallinn University: Tallinna Ulikool, ESTONIA

## Abstract

The aim of this study was to develop and evaluate an instrument to assess international students’ perceptions of the international learning environment called ‘Measure of the International Learning Environment Status’ (MILES). We based the development of the MILES on a solid theoretical framework from Moos by addressing three domains to measure the quality of the international learning environment, namely goal direction, relationships, and system change and system maintenance. We have designed and constructed the instrument in three steps. Firstly, we have collected items from relevant existing instruments and grouped them into the three domains via content analysis. Secondly, we applied a Delphi procedure involving international higher education experts from different stakeholder groups and from different cultural backgrounds to identify and reach consensus on the items comprehensively covering important elements of the international learning environment. Thirdly, we carried out an initial questionnaire evaluation. The final MILES consisted of 47 items with 13 in the first domain, 17 in the second and 17 in the third domain. The content of the domains was clearly in line with Moos theoretical framework and we interpreted the sets of items as goal direction, relationships, and supporting services, respectively. This study provides a comprehensive and systematically developed instrument for future research to better understand international students’ perspectives towards the international learning environment that are supported by stakeholders from a range of cultures.

## Introduction

As Higher Education Institutions (HEIs) recruiting international students have a responsibility to provide international students with optimal support, it is important to have a good understanding of their needs and expectations regarding their international learning environment (ILE) [1–4]. In acknowledging the importance of hearing international students’ voices, their needs, expectations, and experiences have been investigated from various angles, including their academic and cultural challenges [1,5], adjustment and adaptation processes [6,7], and satisfaction with their host institution’s services [2,8,9]. A previous study observed that individual HEIs addressed different aspects while aiming for the same aim, namely understanding international students’ experiences [10]. Therefore, the HEIs may not comprehensively address all essential needs, expectations, and experiences of international students regarding their ILE and, hence, miss out on important information. To help HEIs gain a holistic understanding of the needs, expectations, and experiences of their international students and to be able to provide a conducive ILE, we aimed to conscientiously develop a tool covering all essential elements of the ILE.

Applying a solid theoretical framework could enable HEIs to obtain an encompassing picture of the ILE quality. Besides, it may help improve the quality of further research as it enables researchers in related fields to build on each other’s work [11–13]. A decade ago, Schönrock-Adema and her colleagues attempted to find solid theoretical frameworks among educational environment research and identified two underlying conceptual frameworks, one from Murray and another from Moos [13]. The variables and concepts from Murray’s framework were unstructured and not univocal [13]. By contrast, Moos’ theoretical framework could provide a systematic theory and central guidance for learning environment research [13]. Moos summarized three broad domains applicable to any type of human environment that are critical in determining the quality of those environments, including personal development or goal directions, relationships, and system maintenance and system change [14–16]. These three domains can be integrated into various settings such as family environment and child development, healthcare setting and patient improvement, and educational environment and student growth, thus providing comprehensive coverage and reflection for each setting [13–19]. Furthermore, the Moos’ theoretical framework has been widely applied to the development of survey instruments in certain environmental contexts as a checking lens for scale classification, item comparison, and coverage examination [17–20].

Taking a closer look at these three broad domains and interpreting them in the context of the learning environment, reveals the following characteristics: (1) The personal development or goal direction domain relates to the underlying goals of the particular environment. Personal growth and self-improvement often take place along these directions. For learning environments, it usually means the learning objectives, content, and constructive criticism [13]. (2) The relationships domain relates to the extent of people being involved in their environments, supporting and helping each other, and expressing themselves spontaneously. The characteristics of conducive learning environments are open communication, sense of belongings, friendliness, and social and interpersonal support [13]. (3) The system maintenance and system change domain relates to order, organization, clarity of rules, and reactivity to challenges. In learning environment settings, this frequently means facility construction, teacher control, response to student perceptions and innovation [13]. These three domains together provide categories of important elements for quality assurance of the learning environment and leave room for further research into interpretations that are specifically integrated with certain contexts [13–16].

Although there are already instruments for evaluating the quality of the learning environment in general that have been based on the framework of Moos, we do not consider these instruments as fully applicable to the *international* learning environment because international students may have specific needs with regard to, for example, the learning content and the supporting services [10,21,22]. International students may, for instance, need help due to cultural differences between their home country and the host country [21]. Meeting international students’ needs and understanding their perceptions of the ILE could not only optimize students’ wellbeing [6,23,24], but also benefit HEIs by preventing drop-out [25], improving educational outcomes, and enhancing competitive advantages of attracting students [21,26]. The aims of this study were to develop a theory-based and comprehensive ILE evaluation questionnaire for the *international* learning environment—the Measure of the International Learning Environment Status (MILES)—that can be applied to different cultural contexts and to perform a first questionnaire evaluation.

## Methods

**Fig 1:**
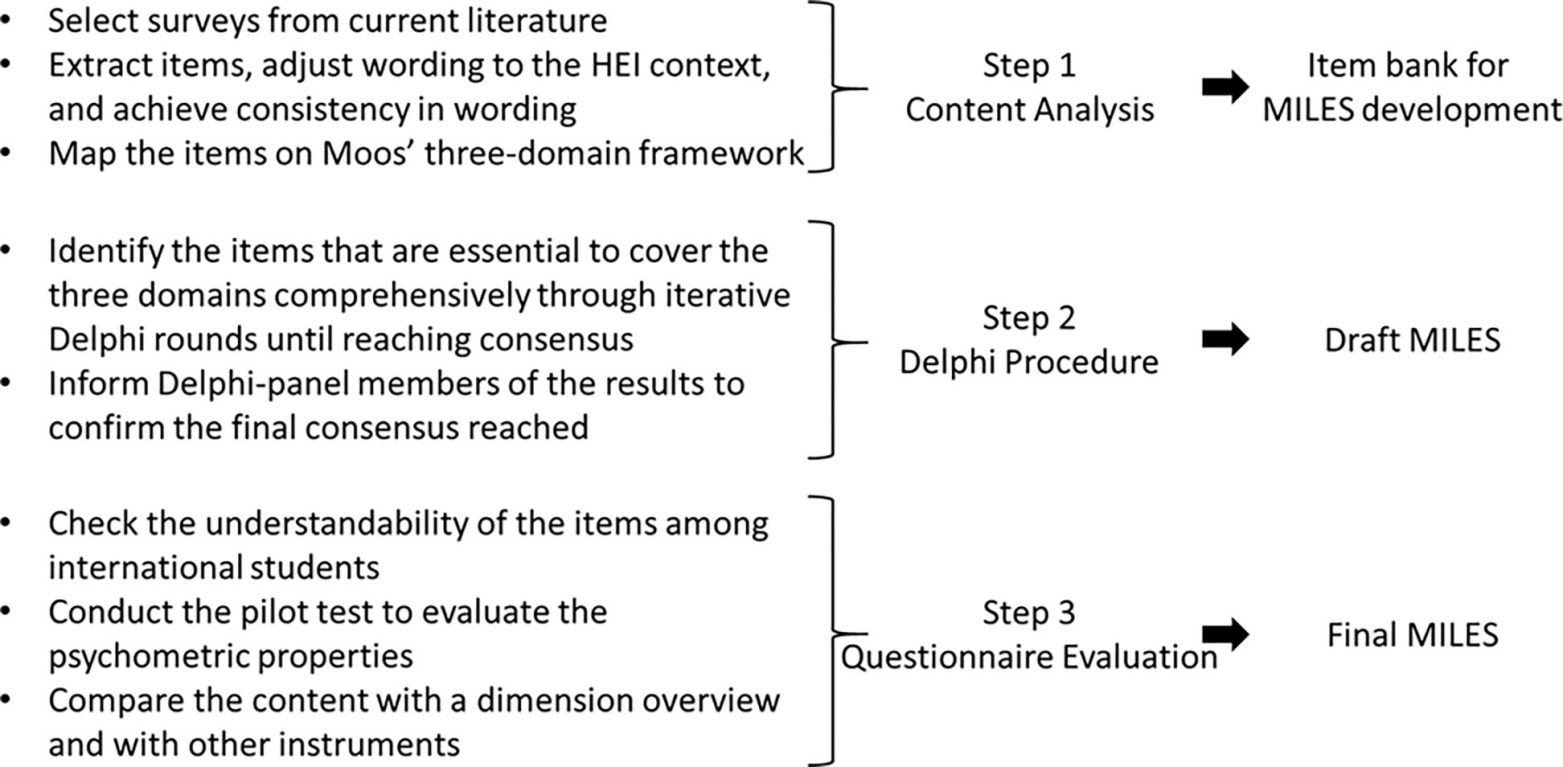
Overview of the development process of the MILES.

We defined international students as students who move to a country other than their country of origin for the purpose of study. We constructed the MILES in three steps: (1) a content analysis to collect items from relevant existing instruments that had been retrieved from a systematic review [10] and to group them into Moos’ three domains; (2) a Delphi procedure to identify and reach consensus on the items needed to comprehensively cover important elements of the ILE; (3) a first questionnaire evaluation. Fig 1 shows an overview of the questionnaire development process. The steps we took align with the AMEE Guide for developing questionnaires for educational research [27]. The process and the results of each step are explained in the following sections.

### Content analysis

Based on a systematic review of studies focusing on international students’ needs, expectations and experiences of their ILE [10], all studies describing the development or application of instruments relevant to the evaluation of the ILE were identified. We took this subset of studies as the starting point for our analysis and screened the instruments for their relevance. We included instruments that were (1) designed to evaluate international students’ needs, expectations and experiences in HEIs as well as (2) published in peer-reviewed journals. We excluded instruments that (1) focused on a particular academic discipline, such as engineering education or medical education; (2) focused on a specific aspect of internationalization of higher education, such as international students’ interaction needs with domestic students; or (3) did not contain complete sentences. From the remaining instruments, we extracted those items that concerned international students’ perceptions of their international higher education learning environment. We deleted duplicate items and merged near-duplicate items. Thereafter, we screened the extracted items to check whether their content was relevant or applicable to the evaluation of the quality of the ILE, and, if necessary, reformulated the items to make their wording consistent while keeping the core content intact. For instance, we adjusted the original items “*The University I have chosen has good access to computer labs*” and “*My university has good learning resources*” into “*This higher education institution has good access to computer labs*” and “*This higher education institution has good learning resources*”, respectively, to have consistent wording in our item bank.

Subsequently, we performed a content analysis on the items: five higher education researchers (*JSA*, *NAB*, *MHP*, *RD*, *XX*) who were all familiar with Moos’ theoretical framework independently mapped the items on Moos’ three-domain framework. They assessed in which domain(s) an item fits best and ticked one or more domain(s) accordingly. If they considered an item as not representative of any of these domains, they ticked “none of all”. If three or more researchers (≥ 60%) assigned an item to the same domain, we considered its connotation univocal and placed the item in that domain. We split and reformulated items that were considered as representing more than one domain in such a way that they belonged to only one domain and repeated the content analysis process for these items. The results from this step formed an item bank for the development of the MILES.

### Delphi procedure

In the second step, we applied a modified Delphi procedure to identify the items to be included in the MILES. Our Delphi study involved key stakeholder groups of international higher education institutions delivering international education, which comprised teachers/staff, international students, policy makers/advisors, and researchers. The Delphi panel members could indicate that they belong to more than one stakeholder group. We recruited the Delphi panel members from our own university through inter-departmental international classroom events and globally by contacting authors who had already performed related research in the field of internationalization in higher education. We asked the corresponding authors to recommend 2–3 stakeholders (they could be one of the stakeholders). We aimed to recruit 16–24 participants to join the Delphi panel and strived to maintain a balanced composition of stakeholder groups, with no less than 25% of each stakeholder group. Where necessary, we used a snowballing approach to increase the number of specific stakeholder groups by contacting the initial group of stakeholders [28].

Hereafter, we performed the first round of the Delphi survey asking the Delphi-panel members to select–per domain–the eight items from the items bank that they considered the most important and which, taken together, provided comprehensive coverage of the domain, and to rank these items by order of importance. Items that were selected by at least four participants were included in a first draft instrument. This first draft instrument was presented to the Delphi-panel members in the second round, along with additional lists with items that were selected as the top 8 by two or three participants, or as the top 3 by a single participant. In the second round, we asked the participants to select no more than three items per domain from the additional list based on their perceived importance and on the degree to which the domain was covered comprehensively. Items that were selected by four or more participants in this round were added to the draft instrument. We also asked participants open-ended questions to check for additions, deletions, or comments. In the next rounds, this process (in which we asked the participants to select once more a maximum of three items per domain from the additional list) was repeated until consensus was reached. Afterwards, we informed Delphi-panel members of the results to confirm the final consensus reached. The result of this step was the draft MILES.

### Questionnaire evaluation

After constructing the draft MILES, we applied it in practice to evaluate the understandability of the items, to investigate its psychometric properties, and to examine the comprehensiveness of the content. First, we sent the draft MILES to several international students (n = 8) to check the understandability of the items. Participants completed the questionnaire and, in case they found items unclear, provided suggestions for improving the wording. After making any necessary changes, we held group interviews to check whether our modifications to the MILES led to improvements.

Then, we sent the revised MILES to international diploma-seeking students from different faculties at the University of Groningen via Qualtrics (n = 100) to collect data, so that we could examine the psychometric properties of the MILES. Similar research methods has been used in a former study, in which the sample size of per dataset was 104 participants [19]. Besides, the sample size of the instruments on which our questionnaire was based ranged from 51–710. Thus, we considered the sample size of 100 to be sufficient in our pilot test for the MILES. Descriptive statistics of the respondents are shown in Table 1. Sixty-two (62%) participants were bachelor students and 38 (38%) were master students. Female students accounted for 72% of all participants. Most international students reported that they belonged to the Germanic Europe cultural group (37%), followed by the Eastern Europe cultural group (25%). Fifty-four percent of our participants were medical students. We asked the participants to score each item on a 5-point Likert scale, ranging from 1 (completely disagree) to 5 (completely agree). The questionnaire can be completed in 10–15 minutes. We pilot tested the MILES to evaluate its reliability by calculating Cronbach’s α (≥0.9–Excellent;≥0.8–Good;≥0.7–Acceptable;≥0.6–Questionable; ≥0.5–Poor;<0.5–Unacceptable) [29,30]. Moreover, we omitted the item if its “Cronbach’s α if Item Deleted” would result in a reliability improvement [31]. We used SPSS 26 to conduct all the analyses.

**Table 1:** Descriptive statistics of the pilot test participants.

	Number	Percent (%)
**Study Stage**		
Bachelor	62	62
Master	38	38
Total	100	100
**Study Year**		
First-year	45	45
Second-year	27	27
Third-year	23	23
Fourth-year and above	5	5
Total	100	100
**Gender**		
Male	26	26
Female	72	72
Non-binary / third gender	1	1
Prefer not to say	1	1
Total	100	100
**Cultural Groups** (*participants could choose more than one cultural group they belong to*)		
Eastern Europe	25	25
Germanic Europe	37	37
Latin Europe	12	12
Nordic Europe	10	10
Middle East	10	10
Anglo	9	9
Southern Asia	9	9
Confucian Asia	9	9
Sub-Saharan Africa	4	4
Latin America	4	4
Total	100	100
**Faculty of Studying**		
Medical Science	54	54
Faculty of Law	18	18
Faculty of Economics and Business	11	11
Faculty of Science and Engineering	8	8
Faculty of Arts	4	4
Behavioral and Social Science	2	2
Campus Fryslân	2	2
Faculty of Spatial Science	1	1
Total	100	100

As a further evaluation of the content validity of our questionnaire, we compared the content of the MILES with the dimensions identified in previous research [10]—language proficiency, academic competence, personal growth, intercultural competence, professional development; relationships with peer students, relationships outside study, relationships with teachers and staff, inclusion in communities, establishing social networks; academic resources, social-cultural resources, facilities and services, career support, initial transition support, psychological support, reputation, and physical safety—provided that the results of the content analysis confirmed them to be appropriate for measuring the quality of the ILE. Additionally, we evaluated the composition of the items in our questionnaire by comparing it with the existing instruments.

### Ethics

Ethical permission was obtained from the Central Ethical Review Committee of the University Medical Center Groningen (CTc UMCG) for the Delphi procedure (Research Register number: 202000483) as well as for the questionnaire application (Research Register number: 202100345). All participants provided informed consent.

## Results

### Content analysis

We included eight instruments comprising a total of 282 items (Table 2). We excluded 84 items that either did not relate to the ILE or were duplicates, which resulted in a total of 198 items remaining for the content analysis.

**Table 2:** Existing instruments used for content analysis.

	Authors (Year)	Region	Content of the instrument	Scales		Number of Items	Design fundamentals (if any)
1	Arambewela and Hall (2006) [32]	Australia	Post-Choice satisfaction of International Postgraduate students studying in Australia		Reliability, Responsiveness, Assurance, Empathy, Tangibles	35	SERVQUAL by Parasuraman, Zeithaml, and Berry [33–35]
2	Chen and Yang (2014) [36]	The U.S.	International students’ striving and thriving experiences in American universities		Academic experience, Socialization experience, Navigation among different culture, Support and assistance from university, Strategies in dealing with challenge	44	
3	Elturki et al. (2019) [37]	The U.S.	International undergraduate and graduate students’ needs and specifically their academic and sociocultural experiences in pathway programs in the United States		Knowledge on language and academic competence, Personal and sociocultural aspects of learning, Academic challenges, Sociocultural challenges, Frequency of seeking support services	55	
4	Gatfield, Barker, and Graham (1999) [38]	Australia	Measuring student quality variables and the implications for management practices in higher education institutions: an Australian and international student perspective		Academic instruction, Campus life, Guidance, Recognition	25	
5	Gu and Maley (2008) [39]	The UK	The intercultural experiences of Chinese students at British universities, and the pedagogical, sociocultural, and psychological challenges that they have encountered		University, Teachers, Student life, Others	28	
6	Jabbar (2012) [40]	Jordan	The benefits international students gained in personal growth, cultural awareness, knowledge of world affairs, and career enhancement at the University of Jordan		Personal growth, Cultural awareness, World affairs, Career enhancement	39	
7	Pereda et al. (2007) [41]	The UK	Service quality in overseas education: The experience of overseas students		Recognition, Quality of instruction and interaction with faculty, Sufficient of resources, Quality of facilities	18	Service Quality dimensions by Lehtinen and Lehtinen [42]
8	Urban and Palmer (2015) [43]	The U.S.	International students’ perspectives of the value of U.S. higher education		Personal and professional goals for coming to the U.S., Institutional support, Engagement in goal achievement	38	

Based on the content analysis, 174 items (88%) were directly mapped unto a certain domain. Ten items (5%) did not belong to any domain and were therefore excluded. These items were, for example, “*This higher education institution has a high image and prestige within the host country*” and “*A degree from this higher education institution has an excellent reputation in my home country*”, which we considered to be beyond the scope of the ILE in HEIs. Fourteen items (7%) were found to be applicable to more than one domain. Based on further discussion within the research team, we split and reformulated these items and repeated the content analysis process for the resulting items to examine whether they could be assigned to a single domain or had to be excluded.

Ultimately, the content analysis resulted in 193 items in total, which included 82 items in the goal direction domain, 41 items in the relationships domain, and 70 items in the system change and system maintenance domain. Considering that we were able to map the vast majority of the items derived from existing instruments to Moos’ theoretical framework, we considered this framework also applicable to the ILE setting.

### Delphi procedure

After inviting international stakeholders, 22 potential Delphi panel members from the Netherlands, Australia, the UK, Turkey, the U.S., and Malaysia signed the informed consent form and were willing to participate. We needed four rounds to complete the Delphi procedure. There were 17, 14, and 14 participants who accomplished the first, second, and third round of the Delphi procedure respectively. The fourth and final round was a confirmation round. The details of the Delphi panel members in the three survey rounds are shown in Table 3. The first Delphi round resulted in the goal direction, relationships, and system change and system maintenance domains containing 10, 16, and 14 items respectively. Based on the second round, 5 items were added (3,1,1 in the three domains respectively). The third round led to the addition of two more items to domain 3 based on the comments in open questions. In the final round, in which we sent an email to the participants asking if they agreed with the latest version, consensus was reached on this set of items. The result was a draft MILES containing 47 items: 13 items in goal direction, 17 items in relationships, and 17 items in system change and system maintenance.

**Table 3:** Details of the Delphi panel members in the three survey rounds.

	Round 1		Round 2		Round 3	
Stakeholder group	N	Percentage	N	Percentage	N	Percentage
**Teachers/staff:**	10	58.8%	6	42.9%	9	64.2%
**International students:**	7	41.2%	7	50.0%	5	35.7%
**Policy maker/advisor:**	5	29.4%	4	28.6%	6	42.9%
**Researchers**	7	41.2%	4	28.6%	7	50.0%
**Total**	17	100.0%	14	100.0%	14	100.0%

Note: Delphi panel members could choose more than one stakeholder group they belong to.

The most valued item that has been regarded as important by Delphi panel members in the goal direction domain is about being a responsible citizen (8/17 marked it as important in Round 1). In the relationships domain, the most highly valued item is about building intercultural friendships (12/17 marked it as important in Round 1). In the supporting services domain, the most highly valued item is about offering academic support (8/17 marked it as important in Round 1). In addition, the Delphi panel members mentioned two extra items that should be considered when measuring the international learning environment: “At this higher education institution, non-academic (supporting) staff members know and speak English” and “At this higher education institution, international students have opportunities for co-governance, for instance by making the information easily accessible in English”. These two items were not included in the existing instruments and they compensate for the supporting services for international learning environment. We provided detailed three-round Delphi procedure results in the supplementary material (S1 Table).

### Questionnaire evaluation

The consultation of international students to examine the understandability of the items led to the adjustment of the wording of several items. For instance, we modified “*This higher education institution offers students adequate information*” into “*This higher education institution offers students adequate information (such as information for classes*, *study materials*, *social events)*” for better clarification. No item needed to be omitted based on Cronbach’s α if Item Deleted. The final MILES contained 47 items including 13, 17, and 17 items respectively in the three domains (see Table 4). Cronbach’s α of the final MILES was 0.95, with 0.89 for the first domain, 0.88 for the second domain, and 0.90 for the third domain, which means that the reliability of the total final MILES was excellent and that the reliability of each of the subscales was good. We summarized the content of the three domains in the final MILES as ’Goal Direction’, ’Relationships’ and ’Supporting Services’, respectively. The means and Standard Deviations of each item are provided in Table 4.

**Table 4:** Psychometric properties of the final MILES.

	Goal direction (Cronbach’s Alpha: 0.89)	Mean	Standard Deviation	Cronbach’s Alpha if Item Deleted
G1	This higher education institution helped me to become a responsible global citizen, and to see myself as part of an emerging world community, committed to helping build this community’s values and practices.	3.60	1.02	0.88
G2	This higher education institution helped me to use cultural diversity to create new solutions and alternatives	3.35	1.16	0.88
G3	This higher education institution helped me to acquire analytical skills and problem-solving techniques	4.15	0.92	0.88
G4	This higher education institution helped me to develop my ability to adapt to new circumstances and deal constructively with differences	3.86	1.09	0.88
G5	This higher education institution helped me to learn new ways of thinking and acting in my field	4.24	0.88	0.89
G6	The teachers in this higher education institution provide valuable feedback	3.55	1.21	0.88
G7	This higher education institution helped me to develop cross-cultural communication skills	3.49	1.24	0.88
G8	This higher education institution pays attention to considering issues from different cultural viewpoints	3.01	1.33	0.88
G9	The study experience at this higher education institution has taught me how to work in a cross-cultural environment	3.62	1.14	0.88
G10	The teachers teach in an understandable way in class	4.01	0.94	0.88
G11	The courses offered at this higher education institution are appropriate for my needs and aspirations	4.08	0.92	0.88
G12	This higher education institution provides academic courses and training relevant to my future job and career prospects	4.09	1.06	0.88
G13	This higher education institution teaches students the skills necessary for employment	3.53	1.11	0.89
	**Relationships (Cronbach’s Alpha: 0.88)**			
R1	This higher education institution facilitates that students build intercultural friendships	3.44	1.17	0.87
R2	Teachers at this higher education institution encourage their students to work with students from different backgrounds	3.44	1.18	0.88
R3	Teachers at this higher education institution encourage contact among students from different backgrounds	3.20	1.12	0.88
R4	The students in this higher education institution have had opportunities to have serious conversations with students from different backgrounds	3.63	1.12	0.88
R5	At this higher education institution, I feel comfortable to work in groups and share my ideas	3.95	1.02	0.87
R6	This higher education institution offers a comfortable atmosphere that facilitates contributing to class discussions	4.00	0.88	0.88
R7	At this higher education institution, there is a safe climate to ask teachers for help with academic difficulties	3.98	1.10	0.87
R8	At this higher education institution, teachers are willing to help international students with academic difficulties.	3.71	1.11	0.87
R9	This higher education institution offers students the opportunity to meet professionals in the field	3.77	1.16	0.87
R10	The atmosphere at this higher education institution makes me feel safe	3.92	1.06	0.87
R11	This higher education institution encourages close working relationships between students and teachers to ensure appropriate solutions to student problems	3.23	1.09	0.87
R12	This higher education institution encourages domestic students to help their international peer students	2.08	0.98	0.88
R13	At this higher education institution, domestic students are willing to help with my academic difficulties	2.71	1.11	0.88
R14	The environment in this higher education institution is friendly	3.88	0.90	0.87
R15	This higher education institution assisted me in learning how to interact properly with local people	2.75	1.10	0.88
R16	This higher education institution organizes social activities to help international students to get to know domestic students	2.52	1.19	0.88
R17	This higher education institution offers their students opportunities to make friends with other international students	3.54	1.19	0.88
	**Supporting services (Cronbach’s Alpha: 0.90)**			
S1	This higher education institution offers academic support to international students	3.59	1.16	0.90
S2	This higher education institution provides counseling services for students who experience difficulties in their study	3.98	1.03	0.90
S3	This higher education institution has a process to deal with complaints about the adequacy of services and facilities if they occur	3.34	1.09	0.90
S4	This higher education institution maintains high standards of teaching with quality teachers	3.88	.97	0.90
S5	This higher education institution supports international students with orientation programs	3.35	1.12	0.90
S6	This higher education institution has adequate support services available to help international student adjust to the host country	2.86	1.16	0.90
S7	At this higher education institution, teachers reserve enough time for consultation by students	3.18	1.11	0.90
S8	At this higher education institution, I have a feeling of personal safety on campus	4.33	0.95	0.90
S9	This higher education institution provides a systematic educational programme containing a variety of courses	3.99	0.90	0.90
S10	At this higher education institution, the International Student Office provides support for international students	3.37	1.12	0.90
S11	This higher education institution offers support services to help international students handle cross-cultural communication issues	2.93	1.04	0.90
S12	At this higher education institution, the English that the teachers speak is understandable and at an adequate speed	4.13	1.10	0.90
S13	There are clear requirements for each module	3.67	1.13	0.90
S14	This higher education institution offers students adequate information (such as information for classes, study materials, social events)	3.73	1.08	0.90
S15	This higher education institution provides counseling services for students who experience difficulties in living and/or studying	3.39	1.22	0.90
S16	At this higher education institution, non-academic (supporting) staff members know and speak English	4.21	.93	0.90
S17	At this higher education institution, international students have opportunities for co-governance, for instance by making the information easily accessible in English	3.71	1.13	0.90

Subsequently, we compared the content of the MILES with the dimensions identified in a previous study to evaluate the content validity of our questionnaire. Based on the content analysis, we discarded the dimension ’reputation’ as irrelevant for evaluating the content validity of the MILES as its items could not be mapped upon Moos’ three-domain framework and were considered to be beyond the scope of the ILE in HEIs [10,44]. We identified 16 (94%) of the 17 remaining dimensions in the overview. Item about language proficiency has not been included in the final MILES. In total, forty-five of the final MILES items came from the existing eight instruments, and two came from suggestions from Delphi panel numbers. We provided the result of MILES items’ integration with other instruments in the supplementary material (see S2 Table).

## Discussion

The aim of this study was to develop and evaluate an instrument to assess international students’ perceptions of the international learning environment called Measure of the International Learning Environment Status (MILES). We based the development of the MILES on a solid theoretical framework and built it upon prior work using the content of existing instruments that were developed for this purpose. In addition, international higher education experts from different stakeholder groups and from different cultural backgrounds have been consulted to reach consensus on the items that should be included to ensure comprehensive coverage of the essential elements of the ILE. Furthermore, we examined the comprehensibility of the content and investigated the psychometric properties of the MILES by conducting a questionnaire evaluation. This thorough development process resulted in a scientifically sound and comprehensive instrument for evaluating the ILE in different cultural contexts and performing future research.

We have built the development of the MILES on the content of carefully designed, existing instruments published in peer-reviewed journals. Though the existing instruments were intended to measure the same concept, their content differed. Therefore, they were evaluated as not comprehensively addressing *all* important elements of the ILE [10]. By basing our work on both a theoretical framework and the existing questionnaires, we were able—based on the efforts of previous questionnaire developers—to generate a more comprehensive tool for full evaluation of the ILE.

Furthermore, the instruments that we included were developed in different countries and cultures, resulting in an item bank covering various elements. Moreover, the input from experts from different cultural backgrounds and from different stakeholder groups may add to the value of our instrument for having ILEs evaluated in different contexts by students from different backgrounds [45,46]. As students from all over the world may enter the ILE, HEIs need to create an environment that is open and inclusive towards a student body characterized by cultural diversity to be able to understand students’ different perceptions [21,47]. The engagement of international Delphi panel members strengthens the potential of the MILES to be sensitive to the needs of international students from diverse cultural backgrounds. This international panel and the fact that they all agreed with the draft MILES with adding only two new items is promising for its value for a broad range of international HEIs.

Our first investigation into the psychometric properties of the MILES as completed by international students from diverse cultural backgrounds studying at different faculties at an international university in the Netherlands demonstrated good reliability of the final MILES. We further evaluated the content validity by comparing the MILES elements to the overall dimensions [10]. It is noteworthy that no item on language proficiency, a frequently mentioned element in the existing literature, was selected by Delphi panel members. An explanation may be that the experts/stakeholders considered language proficiency as an entering requirement and a communication tool instead of main goal of students’ studying [43,48,49]. Moreover, English as “Medium of Instruction” has been identified as a vital topic and significant trend for the internationalization of higher education [48]. Several items of the MILES have incorporated this element in the "Supporting Services" domain. The rest of the dimensions including academic competence, personal growth, intercultural competence, professional development, relationships with peer students, relationships outside study, relationships with teachers and staff, inclusion in communities, establishing social networks, academic resources, social-cultural resources, facilities and services, career support, initial transition support, psychological support, and physical safety have all been covered by the MILES. Despite the fact that the Delphi panel members had not been informed about the dimensions identified in the systematic review [10], we concluded that the final MILES covered all the important dimensions that pertain to the international learning environment, which can be regarded as support for the comprehensiveness of our questionnaire.

### Strengths and limitations

A main strength of this study is the thorough and systematic development process of the MILES instrument. We based our work on a scientifically sound theoretical framework throughout the questionnaire development process, enabling researchers to communicate about the ILE across different HEIs and even across different learning environments contexts. Recently, a systematic review shows that the theoretical framework of Moos helped us to define three main domains that should be covered in a comprehensive instrument that encompasses all aspects of the international learning environment [10]. Another strength is that including instruments and experts from different cultures may support the applicability of the MILES in diverse cultural contexts. Thirdly, another strength is that we were able to recruit experts for different cultures to participate in the Delphi method. The Delphi panel also consisted of different stakeholders from teaching staff, administration and students. Our results show that we produced an instrument that indeed was found to be more encompassing than existing instruments. The results of this study may remedy the lack of theoretical frameworks in previous studies focusing on international higher education learning environments and provide an instrument for future research to comprehensively evaluate their international students’ perceptions towards the ILE.

A limitation of our study may be that we based our questionnaire development on existing instruments that have emerged from quantitative and mixed methods research, whereas a previous study showed that qualitative research in this field emphasized other aspects, such as relationships [10]. Consequently, we may bear the risk of missing important aspects. However, the use of Moos’ theoretical framework helped us to ensure the thorough coverage of relationships elements. Another limitation could be the analysis of the results of our pilot questionnaire. We limited ourselves to a psychometric analysis where we only calculated the Cronbach alpha and provided the means and standard deviations in this survey. The sample size was limited to 100 respondents as this was shown in different studies to be sufficient for such psychometric analysis. We did not do the factor analysis or correlation to for instance cultural background of the respondents because of the research aim of this study. However, they could be directions for future research to apply the MILES in different higher education contexts.

### Implications for practice and future research

HEIs could use the MILES to gain a comprehensive understanding of their international students’ perceptions and to evaluate and improve their own ILE. The domains of goal direction, relationships, and supporting services could support HEIs to understand in which domain international students need more help and to enhance the quality of their ILE in a more responsive way. Besides, it will be interesting to apply the MILES in different cultural contexts and to compare international students’ different perceptions of their ILEs in order to be able to create the conditions for more multicultural environments. Future research is needed to investigate the relevance of the outcomes of this instrument for daily practice of the HEIs. An interesting option for future research would be to measure the influence of the cultural distance between the involved HEI and the origin of the participation students. Future research could investigate whether and how cultural differences between the international students and the host institutions are of influence on students’ perceptions of the ILE. Besides, it is important to address that using MILES in practice could show a possible bias and whether answering tendencies related to cultural background pose a problem to the use of the instrument.

## Conclusion

Taking a scientifically sound theoretical framework as the lens for our work, we used the content of previously developed instruments to develop a comprehensive instrument for evaluating international learning environments: the MILES. The MILES comprehensively covers the three broad domains of elements that have been found essential to the quality of the learning environment, which we summarized in the ILE context as Goal direction, Relationships, and Supporting services. We included instruments and stakeholders from different countries and cultures to strengthen the applicability of our instrument in ILEs in different cultural contexts and in future research exploring how cultural diversity is influencing the perceptions and experiences of the ILE.

## Supporting information

S1 Table Three-round Delphi procedure results. (PDF)

S2 Table The MILES items’ integration with other instruments. (PDF)
