# Supplementary material for: Development and evaluation of the Measure of the International Learning Environment Status (MILES) in international higher education
Source: PLoS One. 2023 Aug 17;18(8):e0288373. doi: 10.1371/journal.pone.0288373 (PMC10434870; doi:10.1371/journal.pone.0288373)
Supplement: S1 Table — (PDF) [file pone.0288373.s001.pdf]

S1 Table. Three-round Delphi procedure results

			Items	Frequency
Domain 1	Round 1	1	This higher education institution helped me to become a responsible global citizen	8
		2	This higher education institution helped me to use cultural diversity to create new solutions and alternatives	7
		3	This higher education institution helped me to learn new ways of thinking and acting in field	7
		4	This higher education institution helped me to acquire analytical skills & problem solving techniques	5
		5	The teachers in this higher education institution provide valuable feedback	5
		6	This higher education institution helped me to develop cross cultural communication skills	5
		7	This higher education institution helped me to develop my ability to adapt to new circumstances and deal constructively with differences	5
		8	This higher education institution pays attention to considering issues from different cultural viewpoints	4
		9	The teachers teach in an understandable way in class	4
		10	The courses offered at this higher education institution are appropriate for my needs and aspirations	4
	Round 2	11	This higher education institution provides academic courses and training relevant to my future job and career prospects	7
		12	This higher education institution teaches students the skills necessary for employment	4
		13	This higher education institution teaches students how to work in a cross-cultural environment	4
		14	Teachers at this higher education institution are aware of how my cultural background influences my study process	3
		15	This higher education institution helped me to get rid of apprehension and prejudice towards other societies	3
		16	This higher education institution teaches international students to develop networking skills	2
		17	This higher education institution helped me to clearly define personal goals	2
		18	This higher education institution helped me realize that similarities and differences of ideas are equally important	2
		19	This higher education institution helped me to gain more self-confidence	2
	Round 3		*No extra items need to add	
Domain 2	Round 1	1	This higher education institution facilitates that students build intercultural friendships	12
		2	Teachers at this higher education institution encourage their students to work with students from different backgrounds	10
		3	At this higher education institution, there is a safe climate to ask teachers for help with academic difficulties	8
		4	Teachers at this higher education institution encourage contact among students from different backgrounds	6
		5	This higher education institution offers students the opportunity to meet professionals in the field	6
		6	The learning climate at this higher education institution is safe	5
		7	At this higher education institution, I feel comfortable to work in groups and share my ideas	5
		8	This higher education institution encourages close working relationships between students and international student advisers to ensure appropriate solutions to student problems	5
		9	This higher education institution encourages domestic students to help their international peer students	5

		10	At this higher education institution, teachers are willing to help international students with academic difficulties.	4
		11	The environment in this higher education institution is friendly	4
		12	This higher education institution assisted me in learning how to interact properly with local people	4
		13	This higher education institution offers a comfortable atmosphere that facilitates contributing to class discussions	4
		14	This higher education institution organizes social activities to help international students to get to know domestic students	4
		15	This higher education institution offers their students opportunities to make friends with other international students	4
		16	At this higher education institution, domestic students are willing to help with my academic difficulties	4
	Round 2	17	The students in this higher education institution have had opportunities to have serious conversations with students from different backgrounds	5
		18	At this higher education institution, international students meet more often with each other than with domestic students	3
		19	In this higher education institution, I feel comfortable to approach my teachers to ask questions	3
		20	This higher education institution offers me opportunities to get involved in organizations made up with international students	2
		21	Teachers at this higher education institution take care that any discussions in class about certain topics do not offend my culture	2
		22	This higher education institution encourages students to participate in co-curricular activities	2
		23	This higher education institution helped me to develop new professional contacts	2
		24	This higher education institution encourages international students to help their international peers	2
		25	It is easy to make friends with international students in this higher education institution	2
		26	At this higher education institution, other international students are willing to help with my academic difficulties	2
	Round 3		*No extra items need to add	
Domain 3	Round 1	1	This higher education institution offers academic support to international students	8
		2	This higher education institution has a process to deal with complaints about the adequacy of services and facilities, if they occur	7
		3	This higher education institution maintains high standards of teaching with quality teachers	6
		4	This higher education institution offers international student orientation programs as part of student support services	6
		5	This higher education institution has adequate support services available to help international student adjust to the host country	6
		6	At this higher education institution, I have a feeling of personal safety on campus	6
		7	This higher education institution provides a systematic educational programme containing a variety of courses	5
		8	This higher education institution provides counseling services for students who experience difficulties in their study	5

		9	This higher education institution has an International Student Office	5
		10	This higher education institution offers support services to help international students handle cross-cultural communication issues	4
		11	At this higher education institution, the English that the teachers speak is understandable and at an adequate speed without too much accent	4
		12	There are clear and reasonable requirements for each module	4
		13	At this higher education institution, teachers reserve enough time for consultation by students	4
		14	This higher education institution offers students adequate information via the internet	4
	Round 2	15	This higher education institution provides counselling services for students who experience difficulties in living	4
		16	The library facilities at this higher education institution are adequate	3
		17	This higher education institution provides enough practice-oriented education	2
		18	This higher education institution offers opportunities to become acquainted with this region and its people	2
		19	At this higher education institution, the teachers are available for consultation by students	2
		20	The class sizes at this higher education institution are of an appropriate, reasonably small size	2
		21	This higher education institution has an efficient system to assist with student visas	2
		22	The student accommodations at this higher education institution are of a good standard	2
		23	The computer facilities at this higher education institution are adequate	2
		24	This higher education institution has good learning resources	2
	Round 3	+1	At this higher education institution, non-academic (supporting) staff members know and speak English	Confirmed in the final round (Round 4)
		+2	At this higher education institution, international students have opportunities for co-governance, for instance by making the information easily accessible in English	Confirmed in the final round (Round 4)

Table note: Frequency of four and above is the cutting line of whether to include a certain item into the draft questionnaire.
