# Supplementary material for: Development and evaluation of the Measure of the International Learning Environment Status (MILES) in international higher education
Source: PLoS One. 2023 Aug 17;18(8):e0288373. doi: 10.1371/journal.pone.0288373 (PMC10434870; doi:10.1371/journal.pone.0288373)
Supplement: S2 Table — (PDF) [file pone.0288373.s002.pdf]

S2 Table. The MILES items' integration with other instruments

**Items in the final MILES**	Arambewela et al (2006)	Chen & Yang (2014)	Elturki et al (2019)	Gatfield et al (1999)	Gu & Maley (2008)	Jabbar (2012)	Pereda, et al. (2007)	Urban & Palmer (2015)
**Goal direction domain**								
1. This higher education institution helped me to become a responsible global citizen, and to see myself as part of an emerging world community, committed to helping build this community's values and practices.						√		
2. This higher education institution helped me to use cultural diversity to create new solutions and alternatives						√		
3. This higher education institution helped me to acquire analytical skills and problem-solving techniques						√		
4. This higher education institution helped me to develop my ability to adapt to new circumstances and deal constructively with differences						√		
5. This higher education institution helped me to learn new ways of thinking and acting in my field								√
6. The teachers in this higher education institution provide valuable feedback	√						√	
7. This higher education institution helped me to develop cross-cultural communication skills								√
8. This higher education institution pays attention to considering issues from different cultural viewpoints								√
9. The study experience at this higher education institution has taught me how to work in a cross-cultural environment								√
10. The teachers teach in an understandable way in class					√			
11. The courses offered at this higher education institution are appropriate for my needs and aspirations	√			√				

12. This higher education institution provides academic courses and training relevant to my future job and career prospects	√				
13. This higher education institution teaches students the skills necessary for employment					√
**Relationships domain**					
1. This higher education institution facilitates that students build intercultural friendships					√
2. Teachers at this higher education institution encourage their students to work with students from different backgrounds					√
3. Teachers at this higher education institution encourage contact among students from different backgrounds					√
4. The students in this higher education institution have had opportunities to have serious conversations with students from different backgrounds					√
5. At this higher education institution, I feel comfortable to work in groups and share my ideas			√		
6. This higher education institution offers a comfortable atmosphere that facilitates contributing to class discussions				√	
7. At this higher education institution, there is a safe climate to ask teachers for help with academic difficulties		√			√
8. At this higher education institution, teachers are willing to help international students with academic difficulties.					√
9. This higher education institution offers students the opportunity to meet professionals in the field					√
10. The atmosphere at this higher education institution makes me feel safe		√	√		
11. This higher education institution encourages close working relationships between students and teachers to ensure appropriate solutions to student problems	√				
12. This higher education institution encourages domestic students to help their international peer students				√	√

13. At this higher education institution, domestic students are willing to help with my academic difficulties							√
14. The environment in this higher education institution is friendly	√						
15. This higher education institution assisted me in learning how to interact properly with local people				√			
16. This higher education institution organizes social activities to help international students to get to know domestic students		√					
17. This higher education institution offers their students opportunities to make friends with other international students					√		
**System change and system maintenance domain**							
1. This higher education institution offers academic support to international students		√					
2. This higher education institution provides counseling services for students who experience difficulties in their study	√	√					
3. This higher education institution has a process to deal with complaints about the adequacy of services and facilities if they occur	√						
4. This higher education institution maintains high standards of teaching with quality teachers	√			√			
5. This higher education institution supports international students with orientation programs	√						
6. This higher education institution has adequate support services available to help international student adjust to the host country		√					
7. At this higher education institution, teachers reserve enough time for consultation by students			√			√	
8. At this higher education institution, I have a feeling of personal safety on campus				√			
9. This higher education institution provides a systematic educational programme containing a variety of courses				√			

10. At this higher education institution, the International Student Office provides support for international students		√		√		
11. This higher education institution offers support services to help international students handle cross-cultural communication issues	√	√				
12. At this higher education institution, the English that the teachers speak is understandable and at an adequate speed			√		√	
13. There are clear requirements for each module						√
14. This higher education institution offers students adequate information (such as information for classes, study materials, social events)	√					
15. This higher education institution provides counseling services for students who experience difficulties in living and/or studying	√	√				
16. At this higher education institution, non-academic (supporting) staff members know and speak English (*suggested by Delphi panel members*)						
17. At this higher education institution, international students have opportunities for co-governance, for instance by making the information easily accessible in English (*suggested by Delphi panel members*)						
